# Joint Classification of Hyperspectral Images and LiDAR Data Based on Dual-Branch Transformer

**DOI:** 10.3390/s24030867

**Published:** 2024-01-29

**Authors:** Qingyan Wang, Binbin Zhou, Junping Zhang, Jinbao Xie, Yujing Wang

**Affiliations:** 1 School of Measurement-Control and Communication Engineering, Harbin University of Science and Technology, Harbin 150080, China; 2120610164@stu.hrbust.edu.cn (B.Z.); mirrorwyj@hrbust.edu.cn (Y.W.); 2 School of Electronics and Information Engineering, Harbin Institute of Technology, Harbin 150001, China; zhangjp@hit.edu.cn; 3 College of Physics and Electronic Engineering, Hainan Normal University, Haikou 571158, China; jbxpost@hainnu.edu.cn

**Keywords:** hyperspectral image, LiDAR data, transformer, cross modality, feature fusion

## Abstract

In the face of complex scenarios, the information insufficiency of classification tasks dominated by a single modality has led to a bottleneck in classification performance. The joint application of multimodal remote sensing data for surface observation tasks has garnered widespread attention. However, issues such as sample differences between modalities and the lack of correlation in physical features have limited the performance of classification tasks. Establishing effective interaction between multimodal data has become another significant challenge. To fully integrate heterogeneous information from multiple modalities and enhance classification performance, this paper proposes a dual-branch cross-Transformer feature fusion network aimed at joint land cover classification of hyperspectral imagery (HSI) and Light Detection and Ranging (LiDAR) data. The core idea is to leverage the potential of convolutional operators to represent spatial features, combined with the advantages of the Transformer architecture in learning remote dependencies. The framework employs an improved self-attention mechanism to aggregate features within each modality, highlighting the spectral information of HSI and the spatial (elevation) information of LiDAR. The feature fusion module based on cross-attention integrates deep features from two modalities, achieving complementary information through cross-modal attention. The classification task is performed using jointly obtained spectral and spatial features. Experiments were conducted on three multi-source remote sensing classification datasets, demonstrating the effectiveness of the proposed model compared to existing methods.

## 1. Introduction

Remote sensing technology plays an increasingly important role in Earth observation. By analyzing the spectral characteristics of objects in different bands, it is possible to identify, detect changes, and quantitatively analyze land features [1,2]. It has significant applications in fields such as agricultural monitoring, urban planning, military reconnaissance, and others. However, due to the specificity of hyperspectral image classification (classifying each pixel in an image), the impact of cloud cover or shadows during the data collection process is inevitable [3]. This can result in blurred spectral information and inaccurate classification. Additionally, the low spatial resolution exhibited by hyperspectral imagery to some extent limits the overall classification accuracy.

The rapid development of remote sensing sensor technology has made it possible to combine data from multiple sensors to describe land information comprehensively. Data from different sensors provide various types of information about the same geographic area. For instance, hyperspectral imagery effectively captures spectral and spatial information of observed targets [4] and LIDAR utilizes laser pulses to measure the elevation information of the Earth’s surface. The Digital Surface Model (DSM) contains elevation information for each point on the Earth’s surface [5,6,7]. Synthetic Aperture Radar (SAR) uses a radar system to transmit microwave signals, records the returning signals, and then utilizes this data to create high-resolution images. SAR can provide geometric information about surface objects, including their shape, size, and orientation [8]. Therefore, by combining data from different modalities, it is possible to address issues present in a single mode. For instance, combining LiDAR, which is less affected by atmospheric interference and contains rich elevation information, with hyperspectral imagery can provide complementary information [9]. This approach addresses the issue of spectral similarity among different materials by supplementing the spatial information of hyperspectral imagery. Therefore, multiple modalities of data can be used to analyze information related to land cover [10,11]. However, it is essential to address the challenges of disparate information dimensions and unrelated physical features between the two modalities.

In previous research, fusion classification methods for Hyperspectral Imaging (HSI) and Light Detection and Ranging (LiDAR) have often inclined towards reducing data dimensionality and manually designing feature fusion based on the intrinsic properties of the data [12,13,14,15]. For instance, in [14], Liao et al. proposed a method that integrates Morphological Profiles (MPs) of Hyperspectral (HS) and LiDAR data on a manifold using graph-based subspace learning, resulting in improved classification outcomes. In [15], the fusion of Hyperspectral (HS) and LiDAR data was enhanced by using Extinction Profiles (EPs) combined with Total Variation Component Analysis. Additionally, the use of multiple fusion strategies has been proven to further enhance classification performance. For instance, in [16], both feature-level fusion and decision-level fusion were employed, where Gabor features extracted from HSI and LiDAR data, along with their amplitude and phase features, were concatenated and input into the classifier. By normalizing the results of three classifiers from two superpixel segmentation algorithms and adopting a weighted majority voting decision fusion strategy, the efficiency of utilizing multiple features was effectively improved. However, the mentioned approaches heavily relied on manually designed features, incorporating more subjective ideas, making it challenging to adaptively generalize the intrinsic features of multimodal data. Secondly, these traditional methods have not fully exploited spatial information, limiting their classification performance. Moreover, due to the relatively large number of features extracted from different remote sensing data, it may lead to the “curse of dimensionality” problem, where the high dimensionality of features makes processing and analysis complex and challenging. Therefore, while traditional methods have achieved some success in land cover classification accuracy, their applicability and adaptability still need further expansion and improvement.

The algorithm based on deep learning demonstrates significant potential in the joint classification of multi-source remote sensing data [17,18,19]. Chen et al. [20] independently extracted features from multimodal data using a dual-branch CNN, and fused the heterogeneous features of each branch through a fully connected DNN. Building upon a dual-branch deep CNN structure, Xu [21] supplemented spatial information from other modalities in a cascading manner. However, the model does not place sufficient emphasis on spectral features, leading to incomplete feature fusion. Hang et al. [22] proposed a coupled CNN network that optimizes the fusion of multimodal features by combining feature-level fusion and decision-level fusion strategies, resulting in improved classification performance. CNN excels in handling spatial features; however, for HSI data containing a large number of spectral sequence attributes, CNN struggles to identify subtle spectral differences between pixels, especially the mid-to-long-term dependencies between spectra [23]. While Recurrent Neural Networks (RNNs) can establish sequence models, their inability to simultaneously train multiple sample networks limits classification performance.

In order to effectively highlight the key features of each modality and suppress irrelevant information during the analysis, researchers have incorporated attention mechanisms within the CNN framework. This approach is particularly suitable for handling spatial and spectral data, allowing simultaneous analysis of critical components in both types of data. Through attention mechanisms, CNN can focus more on important features in the data while disregarding information that is unimportant or irrelevant to the current task. The Squeeze-and-Excitation Networks (SE) module adjusts channel feature responses to enhance the network’s representational capability [24]. The SE module models interdependencies between channels and adaptively recalibrates channel feature responses, thereby improving the network’s performance significantly. This has led to a notable enhancement in the performance of existing deep learning architectures. Building upon this, Xu et al. proposed a novel multi-scale feature extraction module, SE-Res2Net. It utilizes channel grouping techniques to extract multi-scale features from hyperspectral images, achieving acquisition of different granularity receptive fields. This is combined with a channel optimization module to assess the importance of each channel in the feature map [25]. Roy et al. designed an attention-based adaptive spectral-spatial kernel improved residual network, using spectral attention to capture distinctive spectral-spatial features [26]. Gradually, CNN networks based on extracting both spectral and spatial features have been employed for joint classification of hyperspectral images and LiDAR data. Wang introduced non-local operations as a universal basic building block for capturing long-range dependencies, weighting features from all positions and summing them up [27]. Haut et al. proposed a spectral-spatial attention network based on a residual network. By selecting features at both shallow and deep levels, the network obtains more representative and significant features for classifying hyperspectral image data. Spectral and spatial attention focus on highlighting prominent bands and spatial information, respectively [28].

The Transformer model has garnered attention from researchers due to its excellent ability to capture global relationships [29]. Initially proposed for natural language processing, it has later found applications in image processing [30]. Qing et al. [31], leveraging a multi-head attention mechanism, successfully captured spectral relationships in sequences, enhancing the classification performance of HSI. Hong et al. [32] introduced a spectral transformer model that captures spectral features from neighboring configurational bands. However, the mentioned works did not utilize spatial information. Roy et al. [33] introduced a multimodal fusion transformer. This approach initializes the learning embedding with LiDAR data. However, this operation did not fully integrate effective information from both data sources, limiting classification accuracy.

A Transformer encoder based on self-attention mechanisms can learn sequential information from its own data. Meanwhile, cross-attention mechanisms tailored for multimodal data can concurrently consider relationships between two distinct sequences, thereby better capturing their correlations. In contrast to the MFT proposed by Roy [33], researchers like Zhao [34] introduced a cross-modal attention network. This network combines the learnable labels from the hyperspectral image branch with LiDAR data and computes internal attention to achieve complementary information integration. Similarly, Zhang et al. [35] achieved information fusion between two modalities by exchanging cls (class) tokens and introducing a learnable feature fusion method for modality integration. While the mentioned methods effectively leverage cross-attention mechanisms for complementary information integration, the random initialization of cls tokens significantly impacts subsequent attention calculations. In summary, fusion networks based on CNNs combined with Transformer for cross-modal feature interaction may lead to the oversight of crucial shared high-level features in the processing of multimodal data, thereby impacting the comprehensiveness and accuracy of data analysis. Additionally, due to the distinct discriminative capabilities of specific features in each modal data, a significant imbalance among features may arise.

To better integrate features from hyperspectral imagery and LiDAR data and improve classification accuracy, we propose a dual-branch Transformer feature fusion network. This network focuses on the global information of hyperspectral imagery while considering local neighborhood information. Simultaneously, utilizing a cross-attention mechanism highlights features in hyperspectral images using the attention from LiDAR, achieving complementarity between hyperspectral image and LiDAR data features. Features from both modalities are fused for the classification task. The contributions of this paper are summarized as follows:

(1) The proposed dual-branch Transformer feature fusion network can capture features from shallow layers and integrate them into deep features, thereby achieving complementary information between different modalities.
(2) In response to the relatively weak spatial information of hyperspectral images, a Group Embedding Module is proposed to enhance the local information aggregation between different neighborhoods. This module addresses the issue of neglecting the correlation between adjacent keys in the multi-head attention module.
(3) Considering the physical feature differences between modalities, we utilize mutual mapping of features between modalities to achieve global interaction and improve the performance of joint classification.

## 2. Materials and Methods

### 2.1. Dataset Description

This study conducts classification tasks on three publicly available multimodal remote sensing datasets, namely, the Houston2013 dataset [36], MUUFL Gulfport Hyperspectral and LiDAR (MUUFL) [37,38], and the Trento dataset. The following provides detailed introductions to each dataset along with information on the respective classes.

**Figure 1:**
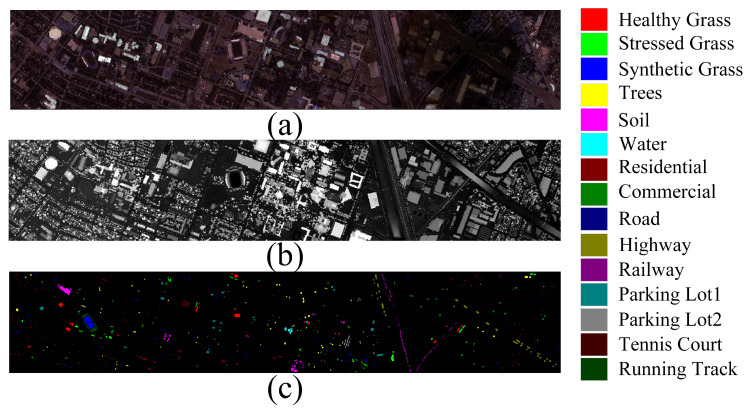
Houston 2013 dataset. (**a**) Hyperspectral image (**b**) LiDAR image. (**c**) Ground truth land cover map.

The Houston2013 dataset is supplied by the 2013 IEEE GRSS Data Fusion Challenge. Gathered in 2012 by the National Center for Airborne Laser Mapping, this dataset comprises topographical details of both the University of Houston campus and the neighboring city. The HSI data consists of 144 spectral bands, while the LiDAR data provides a single band recording elevation information. The image size is 349 × 1905 pixels, with a spectral resolution ranging from 0.38 to 1.05 μm and a spatial resolution of 2.5 m. The dataset comprises 15 land cover categories. Figure 1 displays the pseudo-colored composite image of the HSI data, the grayscale image of the LiDAR data, and the corresponding ground truth map.

**Figure 2:**
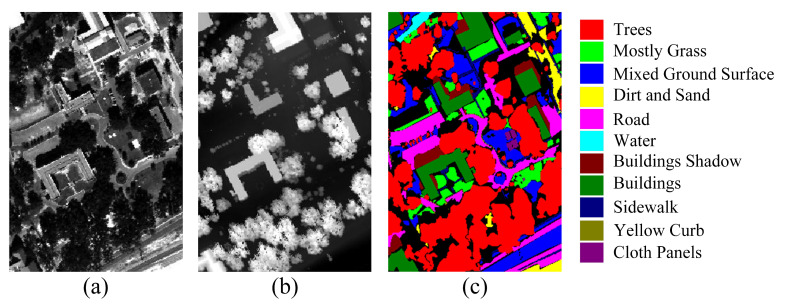
MUUFL dataset. (**a**) Hyperspectral image. (**b**) LiDAR image. (**c**) Ground truth land cover map.

The MUUFL dataset was acquired in November 2010 within the campus area of the Gulf Park campus of the University of Southern Mississippi using the Reflective Optics System Imaging Spectrometer. In the MUUFL dataset, the HSI data comprises 72 spectral bands ranging from 0.38 to 1.05 μm, and the LiDAR data consists of two wavelengths at 1.06 μm. Due to excessive noise, the first 8 and last 8 bands were removed. The dataset consists of 325 × 220 pixels and includes a total of 11 different land cover categories. Pseudo-colored composite images of the HSI data, grayscale images of the LiDAR data, and the ground truth map are shown in Figure 2.

**Figure 3:**
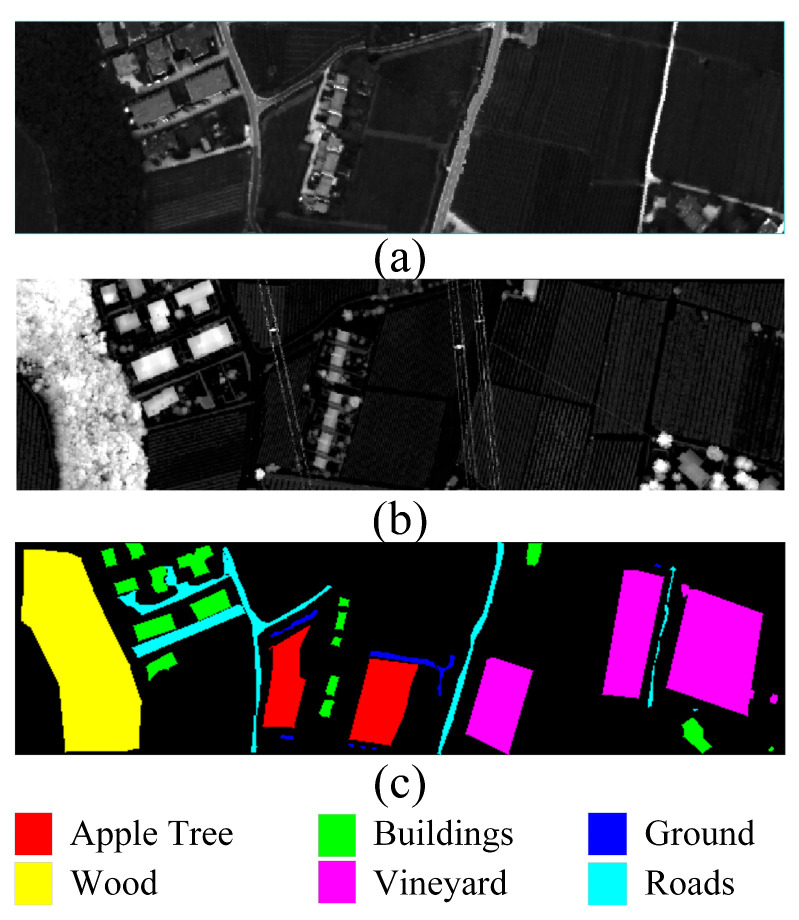
Trento dataset. (**a**) Hyperspectral image (**b**) LiDAR image. (**c**) Ground truth land cover map.

The Trento dataset was collected in southern Trento, Italy, and includes both HSI (Hyperspectral Imaging) and LiDAR DSM (Digital Surface Model) data. The spatial dimensions are 166 × 600, with a spatial resolution of 1 m. The HSI data comprises 63 available spectral bands. The dataset encompasses six object categories, totaling 30,214 sample pixels. Figure 3 displays the pseudo-colored HSI image and LiDAR DSM image of the dataset.

The land cover categories for the three datasets, along with the configuration of training and testing samples, are presented in Table 1.

### 2.2. Methods

**Figure 4:**
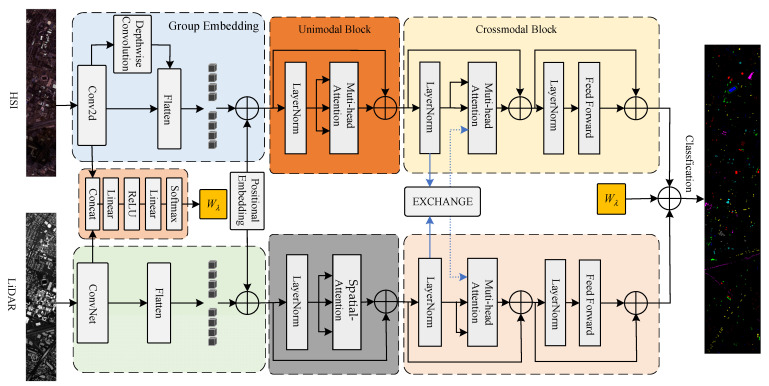
The proposed dual-branch Transformer feature fusion network.

The proposed Dual-branch Transformer feature fusion network is illustrated in Figure 4. The network adopts different processing methods for the information differences between different modalities. It emphasizes spectral features for hyperspectral images and spatial information for LiDAR data. Finally, the information from both modalities is fused for classification.

Based on the outstanding modeling capability of CNN for contextual features, it demonstrates good performance in classification tasks. We first utilize CNN for shallow feature extraction from data of two modalities and control the depth of the output feature maps. Subsequently, we perform feature embedding. This is an indispensable step in entering the Transformer encoding layer.

For different modalities, we undergo distinct serialization processes and then, addressing the characteristics of each modality, respectively enhance the self-attention in different branches of the Transformer layer to extract deep features.

Let HSI be denoted as $X^{H}\in R^{m\times n\times l}$, and LiDAR data of the same geographical area as $X^{L}\in R^{m\times n}$, where m and n represent the spatial dimensions, and l corresponds to the number of spectral bands in HSI. From the normalized data, we construct spectral-spatial cubes $X_{P}^{H}\in R^{s\times s\times l}$ and $X_{P}^{L}\in R^{s\times s}$ for each pixel, where s×s represents the patch size.

To handle pixels at the image boundaries, padding is applied, and the central pixel of each patch serves as a sample label, forming pairs of samples for the two modal-ties.

#### 2.2.1. Feature Extraction from Hyperspectral Image

For hyperspectral images, we employ convolutional layers to locally model the high-dimensional spectral information of HSI, reducing the dimensionality of the spectral information while maintaining the consistency of the sequence length. Here, we set the sequence length to 64, resulting in an output layer size of (s,s,64).

**Figure 5:**
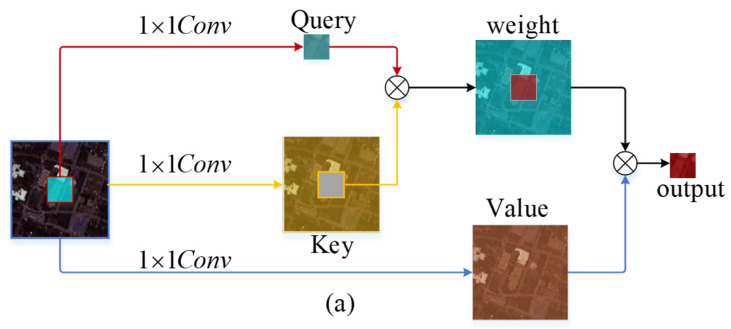

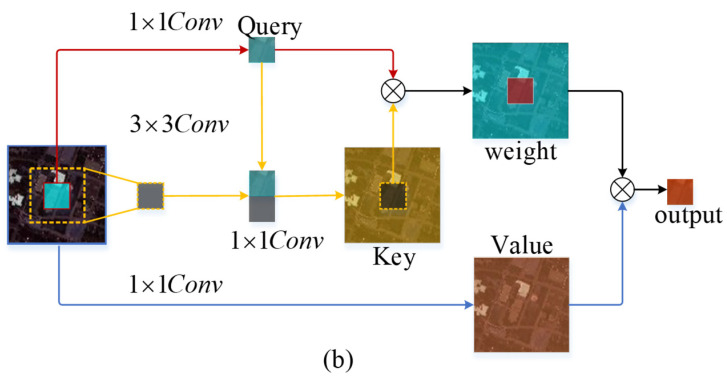
Improvements and differences between enhanced GEM and self-attention: (**a**) Self-attention module computation flow, (**b**) Calculation process of the Group Embedding Module incorporating neighborhood information.

When using one-dimensional positional encoding, the Transformer encoder may lose some spatial information, making it challenging to directly capture the positional relationships of data in a two-dimensional space. In the process of self-attention computation, the rich contextual information between neighboring keys is not fully utilized. Therefore, to address high-spectral images, we introduce a Group Embedding Module (GEM). The computational diagram is shown in Figure 5. This module leverages neighborhood information among input keys to guide self-attention learning. Firstly, GEM captures static spatial contextual relevance among adjacent keys, focusing on the layout or feature distribution of nearby keys in the input. Subsequently, weight coefficients are generated through convolution with queries to explore dynamic spatial contextual relevance. The specific computational process is outlined below:

We first transform it into Query (*Q_H_*) and Value ($V_{H}$) through a learnable embedding matrix.

(1) $$Q_{H},V_{H}=Conv(X_{H}W_{q}),Conv(X_{H}W_{v})$$

where $W_{q},W_{v}$ is a learnable embedding matrix. Unlike the 1×1 convolution used in self-attention mechanisms to generate Key (K), GEM employs a k×k channel convolution to extract spatial neighborhood information, obtaining $K*\in R^{s\times s\times 64}$, which reflects contextual information between neighborhoods. Subsequently, K* is concatenated with Q, and the attention matrix is computed through two 1×1 convolutions.

(2) $$K_{H}=[K*,Q]W_{\theta }W_{\delta }$$

The resulting attention matrix $K_{H}$ obtained in this way contains rich contextual information, unlike traditional attention mechanisms where the attention is isolated to Query-Key pairs. Subsequently, self-attention computation is carried out.

(3) $$Attention(Q_{H},K_{H},V_{H})=Soft\max(\frac{Q_{H}K_{H}^{T}}{\sqrt{d_{k}}})V_{H}$$

By introducing GEM, we incorporate local correlations, while the depth wise convolution captures local spatial information. Combined with the global correlations of the Transformer, this strengthens the model’s capacity to effectively capture HSI data.

#### 2.2.2. Feature Extraction from LiDAR Images

Regarding LiDAR data, we use two 2D convolutional layers to extract its elevation information. The input LiDAR data tensor of size undergoes convolutional operations with 32 and 64 filters, each with a size of 3×3. The convolutional layers with padding produce an output of size (s×s×64). Similar to the hyperspectral image, after the convolutional layers, the LiDAR image also generates 64 two-dimensional feature maps. Additionally, for regularization and to expedite the training process, batch normalization and ReLU activation layers are applied after the convolutional layers.

**Figure 6:**
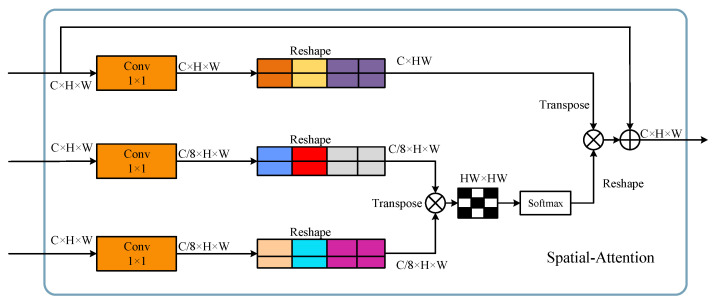
Calculation process of spatial attention. Down-sampling the channels helps to capture the spatial distribution patterns of geographical features more effectively.

Next, it is input into a Transformer encoder based on Spatial Attention (SA). As shown in Figure 6, this attention module is designed to learn representative spatial features by capturing short and long-range pixel interactions from the input feature maps. For an input feature map with dimensions (s×s×64), it is transformed into Query (Q), Key (K), and Value (V) through a learnable embedding matrix.

(4) $$Q_{L},K_{L},V_{L}=Conv(X_{H}W_{q}),Conv(X_{H}W_{K}),Conv(X_{H}W_{v})$$

Through a 1×1 convolutional layer, the channels of $K_{L}$ and $Q_{L}$ are down-sampled by a factor of 8, reducing their channel count to 1/8 of the original. This is done to better capture spatial relationships. By decreasing the channel count, the model focuses more on learning important spatial features. Subsequently, the down-sampled $K_{L}$ and $Q_{L}$ undergo matrix multiplication to form an attention mask of size ss × ss. The attention mask is then subjected to the softmax activation function. The obtained attention mask is multiplied and added to $V_{L}$ in a residual manner, resulting in a spatially attentive output feature map. The final output feature map has dimensions (s×s×64).

Finally, following the same procedure as the HSI processing, attention computation is conducted to complete the aggregation of spatial information.

#### 2.2.3. Feature Fusion of Two Modalities

**Figure 7:**
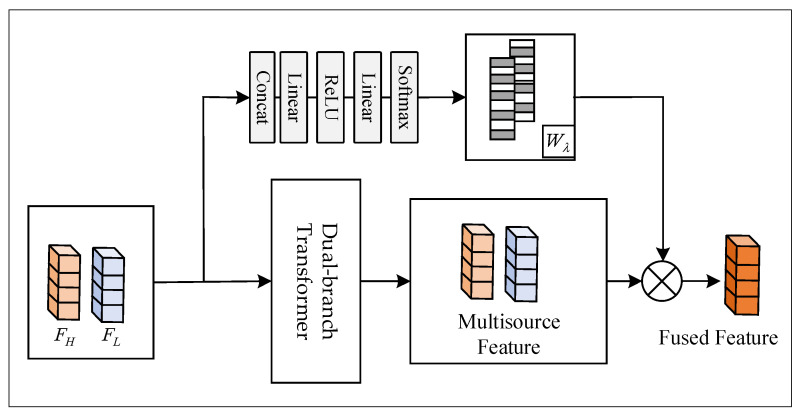
Fusion weight coefficients based on shallow features are used to allocate feature weights for the dual branches.

The extraction of features and the interaction of information in multimodal data are crucial for joint classification tasks. We employ a cross-attention module, allowing the model to weight the features of one modality based on the feature representation of another modality, achieved by exchanging keys between two branches of Transformer layers. By computing attention weights to determine the degree of focus between the two modalities, these weights are then applied to the value vectors of the data, achieving feature fusion and interaction. Leveraging the correlations between different modal data enhances the overall feature representation capability.

(5) $$F=\sum \left[W_{\lambda }MHA(Q_{H},K_{L},V_{H})+(1-W_{\lambda })MHA(Q_{L},K_{H},V_{L})\right]$$

where $Q_{H}$, $K_{H}$, and $V_{H}$ represent the feature embeddings of HSI. $Q_{L}$, $K_{L}$, and $V_{L}$ represent the feature embeddings of LiDAR. $W_{\lambda }$ denotes the weight coefficients, which are obtained through operations such as linear transformations applied to the shallow features of the two modalities, as shown in Figure 7. These weights are used to calculate the fusion weights for HSI and LiDAR data and can be learned and adjusted through parameter updates during the training process. F represents the fused features that enter the classification layer.

The introduction of weight coefficients is due to the unequal importance of hyperspectral and LiDAR data. Hyperspectral imagery occupies the primary features, while LiDAR serves as a supplementary source for spatial information and provides elevation details. After the interaction of information from both modalities, the data proceeds to the classification layer to accomplish the classification task. The following presents the entire algorithmic process of the model (Algorithm 1).
**Algorithm 1** Input: The raw HSI data X_H_, LiDAR data X_L_, and ground truth X_R_
Output: Classification result of each pixel is compared with the overall classification map. 1: Conduct shallow feature extraction on HSI to reduce dimensionality. LiDAR is then mapped to the same dimension as HSI through two-dimensional convolution. 2: Trim datasets for two modalities, dividing them into training sample pairs, validation sample pairs, and test sample pairs. 3: Perform GEM module on hyperspectral data to highlight its spectral information. 4: Perform Spatial Attention to LiDAR data to emphasize spatial information. 5: The cross-attention effectively integrates or aggregates information from two modalities 6: Fusing features using adaptive weight allocation coefficients. 7: Classify the fused features using fully connected layers. 8: Utilizing the trained model to classify the test set and subsequently generate a classification map.

## 3. Experimental Results and Analyses

### 3.1. Experimental Setup and Evaluation Metrics

For the experimental setup, both our method and the comparative methods were executed on the PyTorch 1.10.0 framework under the Ubuntu 20.04 system. The hardware configuration includes an RTX 2080 Ti (11 GB) GPU, a CPU with 12 vCPUs (Intel(R) Xeon(R) Platinum 8255C CPU @ 2.50 GHz), and 40 GB of RAM.

For the network hyperparameters, we set the number of attention heads to 8, and initialized the learning rate to 1.0 × 10^−4^, utilizing weight decay for optimization during training. The batch size during the training phase was set to 64, and the model was trained for a total of 150 epochs. We employed the Adam optimizer for network optimization.

To assess the classification performance of the proposed framework and other existing frameworks, three widely used quantitative analysis metrics were employed: Overall Accuracy (OA), Average Accuracy (AA), and Kappa coefficient (Kappa).

### 3.2. Experimental

To validate the effectiveness of the proposed method, experiments were conducted by comparing it with five other multimodal data fusion classification methods using the same training and testing datasets: EndNet [39], MFT [33], MGA [40], Coupled CNN [22], and HCT [34]. Table 2, Table 3 and Table 4 show the Overall Accuracy (OA), Average Accuracy (AA), Kappa, and class accuracies obtained using different methods on the Houston2013, MUUFL, and Trento datasets.

EndNet adopts an encoder–decoder network architecture, employing a mandatory fusion functionality to sequentially reconstruct multimodal inputs, thereby enhancing cross-modality neuron activation. MFT changes the Transformer’s CLS by incorporating features from one modality, leveraging additional information sources for better generalization, and learning unique representations in a simplified and stratified feature space. MGA utilizes a triple-branch architecture to learn the spectral features, spatial features of hyperspectral images, and elevation information from LiDAR data, respectively. It strengthens the feature interaction of each branch through multi-level feature fusion. Coupled CNN consists of two convolutional neural networks, which are coupled together through a shared parameter strategy. It employs both feature-level and decision-level fusion methods to fully integrate these heterogeneous features. HCT also adopts a dual-branch architecture similar to MFT, fusing multisource heterogeneous information through a cross-token attention fusion encoder.

During the experiment, we randomly selected 50 samples from each land cover type as training samples, with the remaining samples used for testing. Subsequently, training and testing were carried out across various methods, ultimately yielding the classification results for each method. This process was repeated five times, and the final results were obtained by calculating the average.

#### 3.2.1. Setting the Size of Image Patches

**Figure 8:**
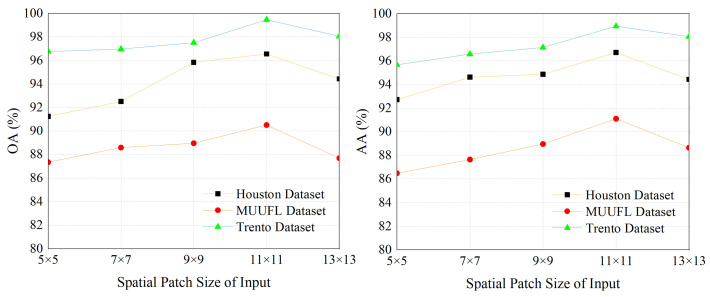
The impact of different spatial patch sizes as network inputs on OA and AA across three datasets.

The patch size will affect the range of the neighborhood that the network attends to around the central pixel. The setting of this parameter is crucial. To find the optimal patch size for our experiments, we conducted trials using five different sizes. As shown in Figure 8, the classification performance on three datasets indicates that, for the proposed network, the best-performing patch size is 11 × 11. Consequently, all subsequent experiments were conducted based on this patch size.

#### 3.2.2. Experimental Analysis of the Houston2013 Dataset

**Figure 9:**
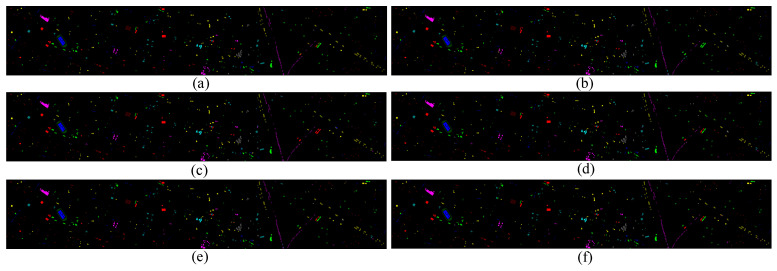
Classification maps by different methods on the Houston2013 dataset (**a**) EndNet, (**b**) MFT, (**c**) MGA, (**d**) CCNN, (**e**) HCT, (**f**) our proposed method.

Table 2 presents the experimental results of the Houston2013 dataset using our method and various comparative methods, including the classification accuracy for each land cover type, the Average Accuracy (AA), as well as the Overall Accuracy (OA), and Kappa coefficient under different classification methods. The results indicate that the final classification accuracy OA increased to 96.55% using the proposed method, and the Kappa coefficient improved to 96.27. Compared to CCNN and HCT, which also employ a dual-branch architecture, the overall accuracy increased by 1.45% and 0.94%, respectively. In fifteen land cover classes, eight classes achieved optimal performance. Figure 9 shows the classification maps of each method, where it is noticeable that Healthy Grass on the right side of the classification map is easily misclassified as Stressed Grass. Due to the dispersed nature of the samples in the Houston2013 dataset and the presence of a lot of background, it is difficult to discern the misclassification in other areas of the classification map. However, in terms of the three performance indicators, the model proposed here outperforms the others.

#### 3.2.3. Experimental Analysis of the MUUFL Dataset

**Figure 10:**
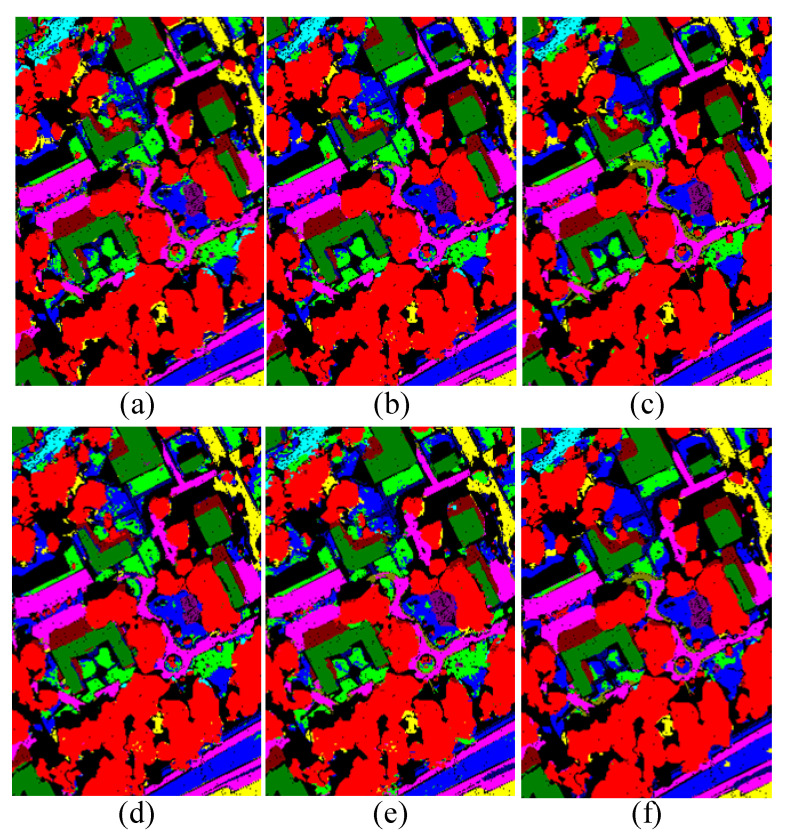
Classification maps by different methods on the MUUFL dataset (**a**) EndNet, (**b**) MFT, (**c**) MGA, (**d**) CCNN, (**e**) HCT, (**f**) our proposed method.

Table 3 presents the experimental results on the MUUFL dataset using our method and various comparative approaches. As shown in the table, the proposed method achieved a final classification accuracy (OA) of 90.51% and a Kappa coefficient of 87.57 on the MUUFL dataset. Among the eleven land cover categories, six categories reached optimal performance. The average accuracy across all categories also reached 91.10%, which is a significant improvement compared to other methods. Figure 10 shows the classification maps for each method, revealing that in the top-right section of the map, despite the presence of numerous region categories, the proposed method still exhibits commendable classification performance, with fewer misclassifications for Mixed Ground Surface. However, the Buildings Shadow category is prone to being misclassified as Mixed Ground Surface. This could be due to the network’s slightly weaker capability to differentiate features between these two land cover types.

#### 3.2.4. Experimental Analysis of the Trento Dataset

**Figure 11:**
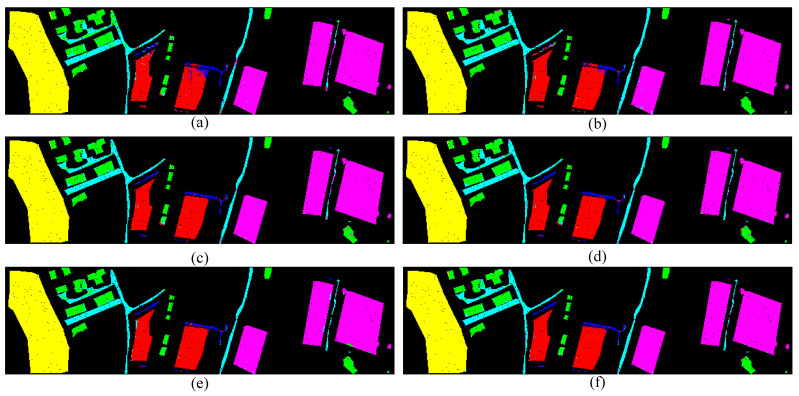
Classification maps by different methods on the Trento dataset (**a**) EndNet, (**b**) MFT, (**c**) MGA, (**d**) CCNN, (**e**) HCT, (**f**) our proposed method.

Table 4 presents the experimental results on the Trento dataset using our method and various comparative approaches. The Trento dataset is overall very orderly, with a regular distribution of land cover types, hence the overall classification performance is generally good. As shown in the table, the proposed method achieved a final classification accuracy (OA) of 99.46% and a Kappa coefficient of 97.67 on the Trento dataset. Among the six land cover categories, three categories reached optimal performance. The average accuracy across all categories also reached 98.94%. From the classification maps (Figure 11), we can roughly observe that the comparative methods often misclassify at the edges of different land cover types, such as Ground being misclassified as Apple Tree in the central part of the map, which is especially evident in the EndNet method. However, the method proposed in this paper shows slightly reduced misclassification at the edges.

Based on the overall analysis of the three datasets, it is observed that the proposed model demonstrates superior performance in terms of Overall Accuracy (OA), Average Accuracy (AA), and Kappa coefficient. Additionally, it is noted that models with a dual-branch processing approach, such as CCNN and HCT, tend to perform better. The lower classification performance of the comparative models can be attributed to the limited number of training samples chosen, lack of utilization of spatial information, or relatively simple fusion strategies.

On the other hand, our proposed model takes into account neighborhood information at each stage and integrates features from both modalities comprehensively. Therefore, even with scattered sample distributions, this model can better differentiate various land cover categories.

Figure 9, Figure 10 and Figure 11 represent the classification results of each model on the test set. Due to the scattered nature of Houston’s test samples, specific differences are not discernible. However, it can be observed from the MUUFL classification map that the proposed models exhibit better performance at the edges of terrain features.

## 4. Discussion

To investigate the advantages of multi-modal joint classification and the contributions of different modules to performance, a discussion will be conducted for the following scenarios.

### 4.1. Impact of Multimodal Data and GEM Modules

To further assess the performance of GEM and the complementary effects between modalities, we conducted comparative experiments using a baseline network that combines CNN with a Transformer encoder. We initially evaluated the classification performance of single-modal data with both a baseline model based on ViT and the currently proposed method. Subsequently, we performed classification experiments using a dual-branch network that fused LiDAR data. Finally, the GEM module was integrated into the dual-branch network for experimentation. The classification performance obtained on three datasets is shown in Table 5.

According to the data presented in Table 5, it is evident that using only LiDAR data for classification tasks results in poor performance. It is not difficult to understand, since LiDAR data only records elevation information of objects, making it challenging to differentiate between different types of objects based solely on elevation information and edge features. This is particularly evident for the Houston2013 and MUUFL datasets, where the overall accuracies are 60.34% and 45.35%, respectively. In contrast, for the Trento dataset with a simpler distribution of objects and concentrated samples, the classification task can be well accomplished using LiDAR data, achieving an overall accuracy of 81.67%.

When comparing solely using hyperspectral images for classification tasks with the proposed network that integrates multimodal features, significant differences are observed. The network exhibits higher overall accuracies by 0.07%, and 0.96% for the Houston2013 and MUUFL datasets, respectively. Therefore, although hyperspectral images, with their rich spectral information, can distinguish object categories, collaborative classification using multimodal remote sensing images has proven to yield a slight improvement in performance, especially in complex scenarios.

Furthermore, by integrating GEM to emphasize spatial relationships within neighborhoods, the proposed network framework’s classification performance is further enhanced. The accuracy on the Houston2013, MUUFL, and Trento datasets reaches 96.55%, 90.51%, and 99.46%, respectively. Simultaneously, both AA (Average Accuracy) and Kappa values also experience significant improvements, confirming the effectiveness of the GEM module.

### 4.2. Impact of Fusion Weight Coefficients

To comprehensively assess the performance of the feature weighting module, comparative experiments were conducted by varying the fusion coefficients. Five sets of manually set hyperspectral weighting coefficients (W) were established as 0.6, 0.7, 0.8, 0.9, and 1 (using only hyperspectral image data). Additionally, a classification task was performed using a learnable fusion coefficient weighting scheme. The detailed classification performance results for each set are provided in Table 6.

Observing the results, it can be noted that with the increase in the weight of the hyperspectral branch, the performance initially shows an upward trend across the three datasets. However, when only hyperspectral images are used, i.e., in the case of single-modal classification, the performance slightly decreases. This phenomenon is more pronounced for the Houston2013 and MUUFL datasets, while the classification performance for the Trento dataset shows less fluctuation. This is because hyperspectral images, due to their rich spectral information, dominate in the classification task, achieving satisfactory accuracy levels. When hyperspectral imaging is combined with LiDAR data for classification, the spatial and elevation information provided by LiDAR complements hyperspectral images, leading to a slight improvement in classification performance. The use of weight coefficients based on shallow features for feature fusion results in optimal performance. Therefore, employing learnable weight coefficients enhances the rationality of feature fusion.

## 5. Conclusions

In this paper, for the joint classification task of hyperspectral imaging (HSI) and Light Detection and Ranging (LiDAR) data, we propose a dual-branch transformer feature fusion extraction network to extract and fuse features from both modalities. This network combines the feature learning methods of Transformers with Convolutional Neural Networks (CNN), fully leveraging their respective strengths.

For data from different modalities, we propose a shallow feature mapping mechanism that reduces the spectral dimension of HSI and allows for better expression of spatial features in LiDAR data.

For HSI, we introduce an improved self-attention method called GEM, which uses the aggregative abilities of convolutional networks to address the loss of positional information caused by Transformer serialization. For LiDAR, we employ a spatial attention mechanism to enhance the expression of its spatial information.

Finally, in contrast to traditional linear fusion methods, we employ cross-attention fusion strategies and dynamic fusion strategies to enhance the complementarity of information from the two modalities. Experimental validation on three multimodal remote sensing datasets confirms the feasibility and effectiveness of the proposed model.

## Figures and Tables

**Table 1:** Land cover categories of the three datasets and the number of training and test samples.

No.	Houston2013			MUUUFL			Trento		
	Class Name	Train	Test	Class Name	Train	Test	Class Name	Train	Test
1	Healthy Grass	50	1201	Trees	50	23,196	Apple Tree	50	3984
2	Stressed Grass	50	1204	Mostly Grass	50	4220	Buildings	50	2853
3	Synthetic Grass	50	647	Mixed Ground Surface	50	6832	Ground	50	429
4	Trees	50	1194	Dirt and Sand	50	1776	Wood	50	9073
5	Soil	50	1192	Road	50	6637	Vineyard	50	10,451
6	Water	50	275	Water	50	416	Roads	50	3124
7	Residential	50	1218	Buildings Shadow	50	2183			
8	Commercial	50	1194	Buildings	50	6190			
9	Road	50	1202	Sidewalk	50	1335			
10	Highway	50	1177	Yellow Curb	50	133			
11	Railway	50	1185	Cloth Panels	50	219			
12	Parking Lot1	50	1183						
13	Parking Lot2	50	419						
14	Tennis Court	50	378						
15	Running Track	50	610						
	Total	750	14,279	Total	550	53,137	Total	300	29,914

**Table 2:** Classification results of different methods for land cover classes in the Houston2013 dataset (best results are bolded).

NO.	Class	EndNet	MFT	MGA	CCNN	HCT	Proposed
1	Healthy Grass	96.84	86.09	**97.58**	92.64	93.92	97.33
2	Stressed Grass	95.18	91.36	85.79	95.87	94.36	**98.92**
3	Synthetic Grass	99.85	99.84	**100.00**	99.39	98.57	99.84
4	Trees	94.55	94.13	**99.83**	96.58	98.26	94.30
5	Soil	**100.00**	95.97	**100**	99.30	99.30	**100**
6	Water	**98.91**	88.36	97.81	90.36	90.25	97.09
7	Residential	95.48	94.90	90.80	94.86	95.78	**96.14**
8	Commercial	97.06	91.12	87.77	92.68	94.27	**97.48**
9	Road	91.84	**94.09**	79.28	90.45	91.76	92.67
10	Highway	76.46	85.98	91.07	95.17	94.37	**95.41**
11	Railway	95.52	89.28	96.96	98.36	97.23	**98.56**
12	Parking Lot1	81.48	**95.94**	88.33	92.01	92.04	91.71
13	Parking Lot2	**100.00**	97.61	95.70	91.86	98.67	91.40
14	Tennis Court	**100.00**	**100.00**	**100.00**	99.68	99.74	**100.00**
15	Running Track	**100.00**	99.50	**100.00**	98.26	99.89	**100.00**
OA (%)	-	93.68	92.89	92.91	95.10	95.61	**96.55**
AA (%)	-	94.88	93.61	94.06	95.16	95.89	**96.72**
K × 100	-	93.16	92.31	92.33	95.11	95.24	**96.27**

**Table 3:** Classification results of different methods for land cover classes in the MUUFL dataset (best results are bolded).

NO.	Class	EndNet	MFT	MGA	CCNN	HCT	Proposed
1	Trees	91.05	87.65	93.47	87.15	89.63	**93.59**
2	Mostly Grass	**89.90**	72.96	74.79	86.58	87.20	79.14
3	Mixed Ground Surface	63.18	68.41	77.38	78.96	79.96	**83.79**
4	Dirt and Sand	97.35	92.00	97.07	93.41	94.71	**97.74**
5	Road	88.53	86.40	88.83	89.76	82.15	**93.29**
6	Water	**100.00**	**100.00**	**100.00**	99.05	99.65	98.55
7	Buildings Shadow	89.69	**91.43**	88.68	90.28	89.12	88.13
8	Buildings	89.70	89.11	90.90	90.21	90.35	**90.64**
9	Sidewalk	76.32	76.77	70.03	78.96	80.27	**82.69**
10	Yellow Curb	**96.24**	83.45	93.98	94.18	94.55	95.48
11	Cloth Panels	99.08	**99.54**	**99.54**	98.46	97.98	99.08
OA (%)	-	86.81	84.19	88.45	87.02	87.38	**90.51**
AA (%)	-	89.19	86.16	88.61	89.72	89.59	**91.10**
K × 100	-	82.77	79.64	84.91	84.65	85.67	**87.57**

**Table 4:** Classification results of different methods for land cover classes in the Trento dataset (best results are bolded).

NO.	Class	EndNet	MFT	MGA	CCNN	HCT	Proposed
1	Apple Tree	88.56	91.26	97.69	**99.27**	98.26	99.10
2	Buildings	87.90	96.59	98.54	96.65	97.61	**98.95**
3	Ground	97.18	95.28	**100**	98.26	98.34	98.23
4	Wood	98.35	97.84	98.86	**100**	**100**	**100**
5	Vineyard	92.53	98.65	99.24	99.86	99.15	**99.96**
6	Roads	86.89	90.96	92.74	96.52	**97.69**	97.40
OA (%)	-	92.80	96.37	98.18	98.42	99.14	**99.46**
AA (%)	-	90.23	94.43	96.44	96.56	98.51	**98.94**
K × 100	-	90.53	92.53	93.56	94.28	96.47	**97.67**

**Table 5:** Classification performance of the three datasets under different cases.

Cases	Houston2013			MUUFL			Trento		
	OA	AA	Kappa	OA	AA	Kappa	OA	AA	Kappa
Only HSI	95.06	94.56	94.10	87.65	87.73	85.12	96.82	97.04	96.28
Only LiDAR	60.34	62.59	60.52	45.35	47.29	45.63	81.67	80.36	80.94
HSI+ LiDAR (No GEM)	95.13	95.56	95.42	88.61	88.29	85.92	96.61	97.16	96.53
HSI+ LiDAR (GEM)	96.55	96.72	96.27	90.51	91.10	87.57	99.46	98.94	97.67

**Table 6:** Classification performance under different weighting coefficients.

W	Houston2013			MUUFL			Trento		
	OA	AA	Kappa	OA	AA	Kappa	OA	AA	Kappa
0.6	89.72	90.43	90.25	79.46	80.41	80.27	96.24	96.57	94.34
0.7	91.96	91.68	91.26	83.24	83.67	83.26	98.63	98.82	96.24
0.8	94.43	93.87	93.96	86.95	87.43	86.87	98.96	99.02	96.85
0.9	96.41	95.24	95.87	87.42	88.21	87.15	99.27	98.56	97.46
1	95.06	94.56	94.10	87.65	87.73	85.12	96.82	97.04	96.28
$W_{\lambda }$	96.55	96.72	96.27	90.51	91.10	87.57	99.46	98.94	97.67

## Data Availability

Data are contained within the article.
